# Impact of non-neuronal cells in Alzheimer’s disease from a single-nucleus profiling perspective

**DOI:** 10.3389/fncel.2023.1208122

**Published:** 2023-06-14

**Authors:** Tra-My Vu, Vincent Hervé, Anosha Kiran Ulfat, Daniel Lamontagne-Kam, Jonathan Brouillette

**Affiliations:** Department of Pharmacology and Physiology, Université de Montréal, Montréal, QC, Canada

**Keywords:** Alzheimer’s disease, neurons, microglia, astrocytes, oligodendrocytes, pericytes, endothelial cells, single-nucleus RNA-seq

## Abstract

The role of non-neuronal cells has been relatively overlooked in Alzheimer’s disease (AD) neuropathogenesis compared to neuronal cells since the first characterization of the disease. Genome wide-association studies (GWAS) performed in the last few decades have greatly contributed to highlighting the critical impact of non-neuronal cells in AD by uncovering major genetic risk factors that are found largely in these cell types. The recent development of single cell or single nucleus technologies has revolutionized the way we interrogate the transcriptomic and epigenetic profiles of neurons, microglia, astrocytes, oligodendrocytes, pericytes, and endothelial cells simultaneously in the same sample and in an individual manner. Here, we review the latest advances in single-cell/nucleus RNA sequencing and Assay for Transposase-Accessible Chromatin (ATAC) sequencing to more accurately understand the function of non-neuronal cells in AD. We conclude by giving an overview of what still needs to be achieved to better appreciate the interconnected roles of each cell type in the context of AD.

## 1. Introduction

The progression of Alzheimer’s disease (AD) leads to behavioral deficits such as memory loss and other cognitive disabilities. To better intervene on these symptoms, the neural correlates underlying these deficits still need to be fully characterized. Since synapse loss and neuronal death are the strongest predictor of cognitive decline in AD (Terry et al., 1991; Selkoe, 2002; Crews and Masliah, 2010; Brouillette, 2014; Alzheimer’s Association, 2015), much work has mainly focused on the impact of neurons in AD pathogenesis. Even the focus of the drug-based clinical trials has been on anticholinesterases and antagonists of the NMDA receptors which regulate neuronal components to prevent acetylcholine degradation and toxicity induced by excessive calcium entry in glutamatergic neurons, respectively (Scheltens et al., 2021). However, the efficacy of these treatments is low due to their symptom reduction being limited to a short time frame and a subpopulation of patients over the course of the disease. It is still unknown if the cellular and molecular changes underpinning neurodegeneration are primarily related to neuronal dysfunctions or derived first from alterations in the other cell types such as the microglia, astrocytes, oligodendrocytes, pericytes and endothelial cells which are critical in the functioning, maintenance, and survival of the neuronal network.

Genome wide-association studies (GWAS) performed over the last few decades have highlighted that many important risk variants such as APOE, TREM2, or BIN1 have high expression levels in non-neuronal cells (Jansen et al., 2019; Wightman et al., 2021). *In vivo* and *in vitro* studies using different amyloid and tau pathology animal models, along with transcriptomic and epigenomic analyses have confirmed the critical role of these non-neuronal cells in the progression of early to late AD. In the recent years, the development of single-cell RNA-sequencing (scRNA-seq) approaches have allowed us to better understand the transcriptomic changes in each of the cell types simultaneously in a non-biased manner (Cuevas-Diaz Duran et al., 2022).

Since neurons have long axonal and dendritic projections that are tangled with each other and with other cell types, it is extremely difficult to dissociate intact neurons without compromising their integrity, especially from archived, frozen post-mortem brain tissue. Thus, instead of using a single cell approach, most studies have opted to profile single nuclei, where changes in the level of mRNA transcripts first occur. Moreover, single-nucleus RNA-seq (snRNA-seq) can now be combined with single-nucleus Assay for Transposase-Accessible Chromatin sequencing (snATAC-seq) to capture the chromatin accessibility profile in individual cells and identify which transcription factors are driving cell fate during AD pathogenesis. The analysis of mRNA level in neural cells has allowed the field to progress tremendously when it comes to understanding the role of different genes in AD. Recently, the characterization of genes associated with non-neuronal cell types and their correlation with AD have been gaining momentum. Here, we reviewed more specifically the different studies that have used snRNA-seq and snATAC-seq to decipher the impact of non-neuronal cells in AD.

## 2. Microglia

**FIGURE 1:**
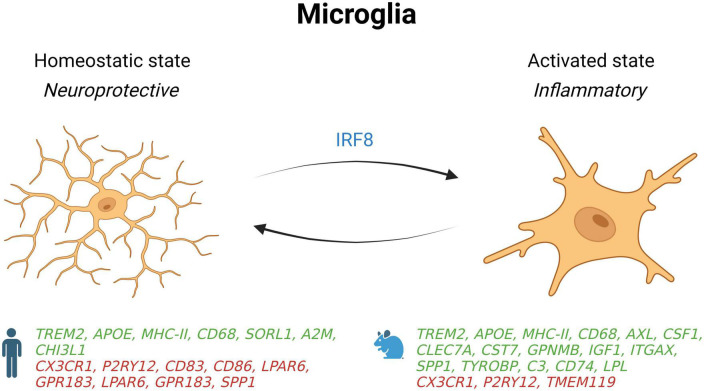
Schematic summary of microglial states in AD patients and mouse models. Genes upregulated (green) and downregulated (red) in snRNA-seq and snATAC-seq studies when comparing AD patients or 5xFAD mice with their respective controls. *IRF8*, interferon regulatory factor 8; *SORL1*, sortilin related receptor 1; *A2M*, alpha-2 macroglobulin; *CHI3L1*, chitinase 3 like 1; *CD68*, *CD74*, *CD83*, and *CD86*, cluster of differentiation 68, 74, 83 and 86; *CX3CR1*, C-X3-C motif chemokine receptor 1; *P2RY12*, purinergic receptor P2Y12; *LPAR6*, lysophosphatidic acid receptor 6; *GPR183*, G protein-coupled receptor 183; *SPP1*, secreted phosphoprotein 1; *AXL*, AXL receptor tyrosine kinase; *CSF1*, colony stimulating factor 1; *CLEC7A*, C-type lectin domain containing 7A; *CST7*, Cystatin F; *GPNMB*, glycoprotein nmb; *IGF1*, insulin like growth factor 1; *ITGAX*, integrin subunit alpha X; *TYROBP*, transmembrane immune signaling adaptor TYROBP; *C3*, complement C3; *LPL*, Lipoprotein lipase; *TMEM119*, transmembrane protein 119.

Microglial account for approximately 10–15% of the cells found within the brain and are the main active immune defense of the central nervous system (CNS) (Dos Santos et al., 2020). They are the first cell population to respond to cellular damage, foreign bodies, or pathogens in the brain to ensure maintenance of the neural tissue (Kreutzberg, 1995). In the homeostatic state, microglia are at rest in a ramified morphology and constantly monitor the cellular environment to act rapidly in response to a pathogen or tissue lesion (Figure 1; Davalos et al., 2005; Nimmerjahn et al., 2005). Upon injury or infection, microglial cells are activated, and their morphologies change to an amoeboid-like form to detect pathogen-associated molecular patterns (PAMPs) and danger-associated molecular patterns (DAMPs) through molecular pattern recognition receptors (PRRs), including Toll-like receptors (TLRs 1–9) (Mishra et al., 2006). Activation of these receptors triggers intracellular signaling cascades to eliminate pathogens or damaged cells and reestablish a homeostatic state (Colton, 2009).

Many microglial phenotypes with different molecular, metabolic, functional, and morphologic characteristics are associated with AD, such as disease-associated microglia (DAM) and dark microglia (Bisht et al., 2016; Keren-Shaul et al., 2017). DAM have been characterized for the first time in 5xFAD mice (Keren-Shaul et al., 2017), a transgenic AD mouse model that expresses five human familial AD-linked mutations in the APP and PSEN1 genes (Oakley et al., 2006). This subtype of microglia is found near amyloid plaques and expresses proteins involved in lipid metabolism and phagocytosis such as CST7 and LPL (Keren-Shaul et al., 2017). DAM also downregulate homeostatic genes such as CX3CR1 and TMEM119, and overexpress pro-inflammatory genes like TNFα, IL-1β, IL-6, iNOS, CCL3, CCL4, CXCL16 (Keren-Shaul et al., 2017). Moreover, this last scRNA-seq study detected an overexpression of AD-related genes such as APOE, TYROBP and CTSD as well as TREM2 that is required in the second stage of DAM activation.

Apart from DAM, another phenotype called dark microglia has also been characterized at the ultrastructural level (Bisht et al., 2016). Although dark microglia share several markers with DAM, their unique properties distinguish them from other microglial phenotypes. Under physiological conditions, dark microglia are rarely present but become more active than normal microglia under chronic stress, aging, fractalkine signaling deficiency (CX3CR1 KOs), and in the APP-PS1 mouse model of AD. They express CD11b and microglia-specific 4D4 when surrounding synaptic elements, and TREM2 when they interact with amyloid plaques to phagocytose Aβ. Further research is needed to decipher the respective role of dark microglia and DAM in AD pathogenesis.

From the inflammatory point of view, activated microglia are found on a broad spectrum of polarization whose extremes are termed activated pro-inflammatory microglia and resting state (homeostatic) anti-inflammatory microglial cells (Prinz and Priller, 2014). Activated microglia are associated with damage induced by inflammation and produce cytokines such as TNF-α, IL-6, and IL-1β, whereas homeostatic microglia express molecules like IL-10 and TGF-β and are linked to tissue repair and removal of cellular debris (Mantovani et al., 2004; Chhor et al., 2013).

Besides their well-known role in the innate immune response, microglia also participate in many other brain processes including synaptogenesis, neurogenesis, myelination, and cerebral vasculature (Lenz and Nelson, 2018). Indeed, microglial cells were found to be involved in synaptogenesis by participating in synaptic pruning through activation of the complement system (Stevens et al., 2007; Schafer et al., 2012). Synapses containing complement C1q and C3 proteins have been shown to be phagocytosed by microglia after recognition of C3 by the CR3 receptor, which is only expressed by microglia in the CNS (Pierre et al., 2017).

Genome wide-association studies (GWAS) performed over the last few decades have highlighted many risk variants in immune-related genes such as TREM2, HLA-DR, and CD33 that are found in microglia (Jansen et al., 2019). Several single-nucleus transcriptomic studies conducted recently also emphasized the critical implication of microglia in AD pathogenesis. Using a snRNA-seq approach on post-mortem prefrontal cortex tissue from individuals with low to high levels of Aβ burden, Mathys et al. (2019) found two main modules containing genes whose expression in microglia correlated with AD pathology, including APOE, TREM2, MHC class II, PICALM, and MEF2C (see their function in Table 1). They also identified AD-associated microglial subcluster overrepresented in AD samples that contained 28 of the 229 genes that have been shown previously to be upregulated in the mouse DAM signature, including APOE, SPP1, and CD74 (Keren-Shaul et al., 2017). Only the microglial subcluster that was the most enriched with DAM markers contained AD GWAS-risk genes whose expressions were downregulated, including the homeostatic gene CX3CR1 and the cell adhesion genes CD86 and CD83 (Mathys et al., 2019).

**TABLE 1:** Function of genes altered in snRNA-seq and ATAC-seq studies.

Gene names	Abbreviations	Changes	Cells	Functions	References
Acyl-CoA synthetase long chain family member 4	ACSL4	↓	oligo	- Synthesis of cellular lipids	Morabito et al., 2021
Alpha-2 macroglobulin	A2M	↑	micro	- Protease inhibitor	Zhou et al., 2020
Apolipoprotein E	APOE	↑ in micro ↓ in astro	micro astro	- Lipid transport, metabolism, and homeostasis - Innate and adaptive immune responses	Keren-Shaul et al., 2017; Mathys et al., 2019; Lau et al., 2020
Beta-2-microglobulin	B2M	↑	micro	- Antigen processing and presentation, and protein refolding	Lau et al., 2020
Bridging integrator 1	BIN1	↑	Oligo astro	- Organization and control of myelination - Major AD risk variant found in many GWAS	Keren-Shaul et al., 2017; Grubman et al., 2019
Cathepsin D	CTSD	↑	micro	- Protein turnover and proteolytic activation of growth factors - Involve in APP degradation following activation by ADAM30	Grubman et al., 2019
C-C motif chemokine ligand 3	CCL3	↑	micro	- Monokine with inflammatory and chemokinetic properties	Keren-Shaul et al., 2017
C-C motif chemokine ligand 4	CCL4	↑	micro	- Monokine with inflammatory and chemokinetic properties	Keren-Shaul et al., 2017
Chitinase 3 like 1	CHI3L1	↑	micro	- Process of inflammation and tissue remodeling	Zhou et al., 2020
CD14 molecule	CD14	↑	micro	- Cooperates with other proteins to mediate the innate immune response to bacterial lipopolysaccharide, and to viruses	Mathys et al., 2019
CD68 molecule	CD68	↑	micro	- Clear cellular debris, promote phagocytosis, and mediate the recruitment and activation of macrophages	Zhou et al., 2020
CD74 molecule	CD74	↑	micro	- Signal transduction, cell-to-cell interactions, inflammation, apoptosis, angiogenesis, cellular self-renewal, and immunoregulation	Mathys et al., 2019
CD83 molecule	CD83	↓	micro	- Regulation of antigen presentation	Grubman et al., 2019
CD86 molecule	CD86	↓	micro	- Regulation of T-cell activation and immune response	Grubman et al., 2019
Collagen type V alpha 3 chain	COL5A3	↓	astro	- Assembly of heterotypic fibers composed of type I and type V collagen	Zhou et al., 2020; Tcw et al., 2022
Complement C1q	C1Q	↑	astro	- Induce A1 neuroinflammatory reactive astrocytes	Liddelow et al., 2017
Complement C1q subcomponent subunit B	C1QB	↑	micro	- Involve in serum complement system - Role in adaptive and innate immunity	Mathys et al., 2019
Complement C3	C3	↑	micro	- Marker of A1 activation	Liddelow et al., 2017
Complement C4B	C4B	↑	astro oligo	- Provide a surface for interaction between the antigen-antibody complex and other complement components factor - Promote Aβ aggregation	Zhou et al., 2020
Contactin 2	CNTN2	↑	oligo astro	- Organization and control of myelination	Grubman et al., 2019
C-X3-C motif chemokine receptor 1	CX3CR1	↑ in human ↓ in mouse	micro	- Immune response, inflammation, cell adhesion and chemotaxis	Keren-Shaul et al., 2017; Zhou et al., 2020
C-X-C motif chemokine ligand 16	CXCL16	↑	micro	- Regulation of cell growth; response to interferon-gamma; and response to tumor necrosis factor	Keren-Shaul et al., 2017
Cystatin-C	CST3	↑	astro	- Inhibitor of cysteine proteinases	Mathys et al., 2019
Cystatin-F	CST7	↑	micro	- Role in immune regulation	Keren-Shaul et al., 2017
EGF like domain multiple 7	EGFL7	↑	endo	- Endothelial cell adhesion angiogenesis	Lau et al., 2020
Erbb2 interacting protein	ERBIN	↑	oligo	- Require for remyelination of axons	Mathys et al., 2019
Fatty acid-binding protein 5	FABP5	↓	astro	- Coordination of lipid and oxidative metabolism with neurons	Zhou et al., 2020; Tcw et al., 2022
Fms related receptor tyrosine kinase 1	FLT1	↑	endo	- Regulation of angiogenesis, cell survival, cell migration, macrophage function, and development of embryonic vasculature	Lau et al., 2020
Glial fibrillary acidic protein	GFAP	↑	astro	- Marker of reactive astrocytes	Leng et al., 2021
HLA class II histocompatibility antigen, DRB1 beta chain	HLA-DRB1	↑	micro	- Central role in the immune system	Mathys et al., 2019
Hypoxia-inducible lipid droplet-associated protein	HILPDA	↓	astro	- Coordination of lipid and oxidative metabolism with neurons	Zhou et al., 2020; Tcw et al., 2022
Inducible Nitric oxide synthase	iNOS	↑	micro	- Pathogen killing and immune-regulatory effects	Keren-Shaul et al., 2017
Interferon regulatory factor 8	IRF8	↑	micro	- Act as a transcriptional activator or repressor - Negative regulatory role in cells of the immune system	Zhou et al., 2020
Interleukin-1 beta	IL-1β	↑	micro	- Pro-inflammatory cytokine	Keren-Shaul et al., 2017
Interleukin-6	IL-6	↑	micro	- Pro-inflammatory cytokine - Role in immunity, tissue regeneration, and metabolism	Keren-Shaul et al., 2017
Leucine rich repeat and Ig domain containing 1	LINGO1	↑	oligo	- Involved in oligodendrocyte differentiation and myelination - Negative regulator of neuronal survival and axonal integrity	Mathys et al., 2019
Lipoprotein lipase	LPL	↓	micro	- Triglyceride metabolism	Keren-Shaul et al., 2017
Lysophosphatidic acid receptor 6	LPAR6	↓	micro	- Lipid metabolism	Grubman et al., 2019
Major histocompatibility complex, class I, E	HLA-E	↑	endo	- Inflammation, innate and adaptive immune systems	Lau et al., 2020
Major histocompatibility complex, class II, DR alpha	HLA-DRA	↑	micro	- Central role in the immune system and response by presenting peptides derived from extracellular proteins	Zhou et al., 2020
Myocyte enhancer factor 2C	MEF2C	↑	micro	- *Trans*-activating and DNA binding activities - Role in hippocampal-dependent learning and memory - Neuronal development, and electrical activity	Mathys et al., 2019
Neurocan	NCAN	↑	astro	- Modulation of cell adhesion and migration	Zhou et al., 2020; Tcw et al., 2022
Nuclear respiratory factor 1	NRF1	↑	oligo opc	- Regulates mitochondrial function - Regulate multiple AD DEGs and genes near AD risk loci in oligodendrocytes	Morabito et al., 2021
Phosphatidylinositol binding clathrin assembly protein	PICALM	↑	micro	- Role in clathrin-mediated endocytosis	Mathys et al., 2019
Purinergic receptor P2Y12	P2RY12	↑ in human ↓ in mouse	micro	- G-protein coupled receptor involves in platelet aggregation	Zhou et al., 2020
Secreted phosphoprotein 1	SPP1	↑ in Mathys et al. (2019) ↓ in Zhou et al. (2020)	micro	- Involve in cell-matrix interaction - Involve in the pathway that leads to type I immunity	Mathys et al., 2019; Zhou et al., 2020
Serpin family A member 3	SERPINA3	↓ in human ↑ in mouse	astro oligo	- Inhibit serine proteases - Marker of reactive astrocytes	Zhou et al., 2020
Sortilin related receptor 1	SORL1	↓	micro	*- Regulation of endosomal traffic and recycling in neurons*	Zhou et al., 2020
Sterol regulatory element binding transcription factor 1	SREBF1	↓	oligo	- Regulation of lipid metabolism and cholesterol	Morabito et al., 2021
Superoxide dismutase 2	SOD2	↓	astro	- Coordination of lipid and oxidative metabolism with neurons	Zhou et al., 2020; Tcw et al., 2022
Transcription factor EB	TFEB	↑	astro	- Regulation of autophagy and lysosome biogenesis that regulates ten other GWAS genes for AD	Grubman et al., 2019
Transmembrane protein 119	TMEM119	↑ in human ↓ in mouse	micro	- Bone formation and mineralization	Keren-Shaul et al., 2017; Zhou et al., 2020
Triggering receptor expressed on myeloid cells 2	TREM2	↑	micro	- Involve in immune response and chronic inflammation - Role in microglial proliferation, survival, clustering, and phagocytosis	Keren-Shaul et al., 2017; Mathys et al., 2019
Tumor necrosis factor	TNFα	↑	micro	- Pro-inflammatory cytokine mainly secreted by macrophages - Involve in cell proliferation, differentiation, apoptosis, lipid metabolism, and coagulation	Keren-Shaul et al., 2017; Liddelow et al., 2017
TYRO protein tyrosine kinase-binding protein I	TYROBP	↑	micro	- Role in signal transduction, brain myelination, and inflammation	Keren-Shaul et al., 2017
Vimentin	VIM	↑	astro	- Maintenance of cell shape and integrity of the cytoplasm, and stabilizing cytoskeletal interactions - Involve in neuritogenesis and cholesterol transport - Marker of DAAs and adult neurogenesis	Lau et al., 2020
Von Willebrand factor	VWF	↑	endo	- Maintenance of hemostasis	Lau et al., 2020

Micro, microglia; astro, astrocytes; oligo, oligodendrocytes; opc, oligodendrocyte precursor cells; endo, endothelial cells; peri, pericytes.

In the CK-p25 AD mouse model, this same subcluster also comprised 35 genes out of 480 that were identified as a microglial signature, such as HLA-DRB1 and MHC class II genes, as well as 49 genes that haven’t been identified previously in AD animal models, including genes coding for the pattern-recognition receptor CD14 and complement component C1QB (Mathys et al., 2019). Thus, this study only found relatively little overlap between microglial genes differentially expressed in human Alzheimer and those previously found in mouse models. The same was also observed in two other studies with post-mortem human brain samples using a snRNA-seq approach or a sequenced RNA from sorted myeloid cells (Del-Aguila et al., 2019; Srinivasan et al., 2020). This discrepancy could be explained by the fact that microglia are quite rare cells in the brain, and that power to detect microglial gene-expression signatures was limited in these human studies. Another reason would be that single-nucleus sequencing is less sensitive than single-cell sequencing to capture human microglial activation since only a very small population of genes previously identified in the mouse microglial response, such as APOE, SPP1, CD74, and CST3, has been shown to be reduced in nuclei compared to whole cells (Thrupp et al., 2020).

To better define microglial activation in AD, whole cells were isolated from fresh autopsy prefrontal cortex samples of individuals with cognitive impairment or AD dementia for single-cell RNAseq analysis (Olah et al., 2020). This represents to date the largest study investigating different transcriptional states of microglia in AD with a total of 16,096 microglia isolated from 17 participants. Although nine distinct transcriptional states were found, more than 80% of microglia fell into two genes clusters that appeared to represent homeostatic cells. In another cluster, microglial genes involved in the cellular response were upregulated, and were found to be more abundant in AD brains than in control samples. Moreover, the DAM-like signature found in 5xFAD mice was found to be scattered among four human microglial subtypes instead of being observed in a single microglial subtype, as one could have expected. Thus, these data suggest that microglial cells mostly stayed in a homeostatic state even in AD brain, and that human and mice microglia respond differently to amyloidosis.

Species differences for microglial gene expression is an important consideration in single-nucleus profiling studies (Chen and Colonna, 2021). In a snRNA-seq study comparing samples from cortices of 5xFAD mice with dorsolateral prefrontal cortex of AD patients, many gene changes were in opposite direction (Zhou et al., 2020). Indeed, while microglial gene-expression profiles from human microglia show an activation of some homeostatic genes such as TMEM119, P2RY12, and CX3CR1, the profiles from 5xFAD mice show that these genes are deactivated. Mechanistically, these profiles show that upregulation of homeostatic genes in human microglia are driven by interferon regulatory factor 8 (IRF8).

Further inter-species microglial differences in gene-expression include the alteration of AD risk genes such as SORL1, CHI3L1, and α-2 macroglobulin in human AD patients, but not in 5xFAD mice (Zhou et al., 2020). Other key DAM genes described in mice were either undetected (CST7 and LPL) or downregulated (SPP1) in human AD microglia. However, some overlap exists, as an increase in TREM2, APOE, MHC-II, HLA-DRA, and CD68 genes are shown in humans and 5xFAD mice. Interestingly, deletion or mutation of TREM2 in both species was sufficient to decrease microglial activation in response to amyloidosis, which may in turn facilitate the progression of AD. More research into different mouse models will be necessary to better explain these inter-species differences.

The brain area might also have an impact on the transcriptomic state of microglia. Indeed, in a study looking at cell-type specific gene-expression in the entorhinal cortices of late-stage AD (Grubman et al., 2019), microglial homeostatic genes were shown to be downregulated instead of being upregulated as seen in the prefrontal cortex of late-stage AD donors like reported in another study (Zhou et al., 2020). In line with other studies (Krasemann et al., 2017; Tcw et al., 2022), Grubman et al. (2019) also found a lower expression of genes involved in cell adhesion and lipid metabolism in AD microglia such as CD83, CD86, and LPAR6.

The distance between microglia and Aβ plaques can also influence the state of these cells. Since DAM have been shown to be concentrated around Aβ plaques (Keren-Shaul et al., 2017), this could explain some of the discrepancies observed in the microglial response detected in 5xFAD mice and human brain studies that did not take into consideration the proximity of isolated microglia to Aβ plaques. Using a state-of-the-art technique to track spatial transcriptomics of individual cells with protein detection in the same sample, it was recently found that 70% of cells within 10 μm of plaques were DAM, while those localized fewer microns away held a more homeostatic profile (Zeng et al., 2023). Moreover, the genes differentially expressed near plaques identified in this study also partially overlapped with plaque-induced genes detected in a previous report using APP*^NL–G–F^* knock-in mice (Chen et al., 2020). Altogether, these results indicate that microglia can adopt many phenotypes with different features to adjust to various pathological conditions such as AD in function of their localization relative to the Aβ plaques to remodel neuronal circuits for maintaining brain homeostasis.

New molecular technologies are also available to characterize AD-associated gene-regulatory profiles at the transcriptomic and epigenomic levels in the different cell types. In a study combining snRNA-seq with snATAC-seq in the same tissue, it was found that two microglial subpopulations more present in the prefrontal cortex of late-stage AD patients had more open binding sites for the transcription factor SPI1 but lower expression of its candidate target genes (Morabito et al., 2021). Since this transcription factor regulate networks of genes differentially expressed in AD and located at known AD GWAS loci, these results suggest that SPI1 acts as a repressor of many AD risk variants in the late stages of the disease (Morabito et al., 2021). In microglia and neurons isolated from dorsolateral prefrontal cortex of late-stage AD donors, another study profiling single-nuclei RNA-seq and ATAC-seq within the same nuclei showed that the transcription factors ZEB1 and MAFB may regulate transcription of approximately half the *cis*-regulatory elements contained in the chromatin that are unique to AD (Anderson et al., 2023). Thus, these novel single-nuclei multi-omics methods offer exciting possibilities for simultaneously studying open stretches of chromatin where key transcription factors might bind nearby genes whose expression is altered in the various cell types of AD brains.

## 3. Astrocytes

**FIGURE 2:**
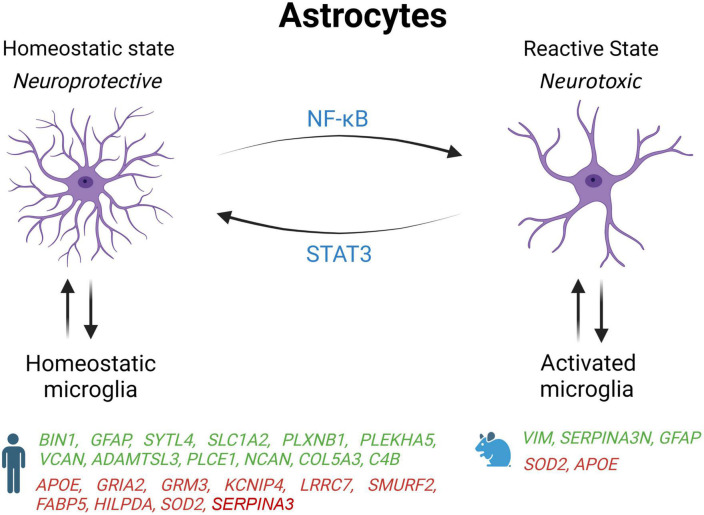
Schematic overview of astrocyte states influenced by microglia activation state in AD patients and mouse models. Genes upregulated (green) and downregulated (red) in snRNA-seq and snATAC-seq studies when comparing AD patients or 5xFAD mice with their respective controls. *STAT2*, signal transducer and activator of transcription 2; *NF*-κ*B*, nuclear factor kappa B subunit 1; *BIN1*, bridging integrator 1; *GFAP*, glial fibrillary acidic protein; *SYTL4*, synaptotagmin like 4; *SLC1A2*, solute carrier family 1 member 2; *PLXNB1*, plexin B1; *PLEKHA5*, pleckstrin homology domain containing A5; *VCAN*, versican; *ADAMTSL3*, ADAMTS like 3; *PLCE1*, phospholipase C epsilon 1; *NCAN*, neurocan; *COL5A3*, collagen Type V alpha 3 chain; *C4B*, complement C4B; ApoE, apolipoprotein E; *GRIA2*, glutamate ionotropic receptor AMPA type subunit 2; *GRM3*, glutamate metabotropic receptor 3; *KCNIP4*, potassium voltage-gated channel interacting protein 4; *LRRC7*, leucine rich repeat containing 7; *SMURF2*, SMAD specific E3 ubiquitin protein ligase 2; *FABP5*, fatty acid binding protein 5; *HILPDA*, hypoxia inducible lipid droplet associated; *SOD2*, superoxide dismutase 2; *VIM*, vimentin; *SERPINA3*, serpin family A member 3.

Astrocytes are the most abundant cell type in the brain that outnumber neurons by more than fivefold (Sofroniew and Vinters, 2010; Freeman and Rowitch, 2013). They perform various functions essential to maintaining homeostasis in the CNS, including preserving the integrity of the blood-brain barrier (BBB), the reuptake of neurotransmitters, synaptogenesis, the production of trophic factors supporting neurons and oligodendrocytes, and the control of the immune system (Barres, 2008; Sofroniew and Vinters, 2010; Araque et al., 2014; Colombo and Farina, 2016). Astrocytes undergo a variety of phenotypic changes in response to a broad spectrum of stimuli. These changes can range from homeostatic to reactive astrocytes (Figure 2; Escartin et al., 2021). Reactive astrocytes increase cell death of neurons and oligodendrocytes and lose their ability to stimulate neuronal survival, synaptogenesis, and phagocytosis of synapses or myelin debris (Liddelow et al., 2017). Reactive astrocytes are highly expressed during neurodegeneration, systemic inflammation, and traumatic brain injury (Liddelow et al., 2017; Yun et al., 2018; Clark et al., 2019; Joshi et al., 2019). Homeostatic astrocytes exhibit neuroprotective actions by increasing tissue repair, neuron and oligodendrocyte survival, and astrocytic glial scar formation (Liddelow and Barres, 2017).

It has also been shown that microglia and astrocytes interact and adjust the neuroinflammatory reaction following injury or disease to preserve brain homeostasis. In response to PAMPs and DAMPs, microglia are activated and adopt a pro-inflammatory profile that can induce the activation of astrocytes (Liddelow et al., 2017). In turn, activated astrocytes produce factors stimulating the activation of microglia thereby inducing a feedback loop amplifying the pro-inflammatory and neurotoxic response. A similar feedback loop exists between homeostatic microglia and astrocytes to promote anti-inflammatory response and tissue repair.

To determine if activated microglia trigger reactive astrocytes in AD, the prefrontal cortices of post-mortem AD tissue was marked with C3, a marker of reactive astrocytes (Liddelow et al., 2017). It was found that 60% of the astrocytes tested positive for C3 in this brain region severely affected in AD. Similar results were also seen in Parkinson’s, Huntington’s, amyotrophic lateral sclerosis, and multiple sclerosis, suggesting that the initial inflammatory response from microglia induces a neurotoxic phenotype in astrocytes which drives neurodegeneration (Liddelow et al., 2017). This study also points out two cytokines (Il-1α, TNF) and one opsonin (C1q) released by microglia that were essential to turn resting astrocytes into a reactive state.

These results are in agreement with another study showing that GFAP, a marker of reactive astrocytes, is upregulated in at least one subpopulation of astrocyte identified by snRNA-seq in two different brain regions: the entorhinal cortex and superior frontal gyrus that are affected early and late in AD, respectively (Leng et al., 2021). Moreover, these cells were expressing fewer markers of homeostatic astrocytes, which support the idea of a transition from a neuroprotective to a neurotoxic status. Interestingly, another single-cell profiling study found two astrocyte subtypes that were observed exclusively in AD brain, whereas the remaining six subtypes were only seen in controls (Grubman et al., 2019). This last study also found that in Alzheimer’s entorhinal cortex, inflammatory genes and TFEB, a key regulator of autophagy and lysosome biogenesis that regulates ten other GWAS genes for AD, are upregulated in specific astrocyte subpopulations driving their transition from healthy to inflammatory states.

Under physiological conditions, astrocytes provide metabolic support to neurons by helping with the storage of fatty acid, and by detoxifying reactive oxygen species (ROS). However, two single-nucleus studies reported that human AD astrocytes decreased the expression of genes involved in the coordination of lipid and oxidative metabolism with neurons, including FABP5, HILPDA, and SOD2 (Zhou et al., 2020; Tcw et al., 2022). Both studies also noticed in AD astrocytes an upregulation of genes encoding proteins of the extracellular matrix such as NCAN and COL5A3, which could be implicated in glial scarring.

One major advantage of using single-cell approaches over the analysis of a mixed population of cells in bulk tissue is the possibility of unmasking differences in gene expression that are regulated in opposite direction in distinct cell types. For instance, the expression of BIN1, an AD risk variant found in many GWAS, was reported to be upregulated in astrocyte but reduced in neurons and not affected in microglia (Grubman et al., 2019). Two single-nucleus profiling studies have also found that the level of APOE transcripts is increased in microglia but decreased in astrocytes from AD brain samples (Grubman et al., 2019; Mathys et al., 2019). Since both human astrocytes and microglia from APOE4/4 carriers have decreased lipid clearance, enhanced cholesterol accumulation, and higher production of extracellular matrix protein and proinflammatory cytokines than their APOE3/3 counterparts (Tcw et al., 2022), this suggest that APOE4 may drive both cell types toward an AD state, especially in microglia that express more APOE in the context of AD. Since APOE4 is the greatest genetic risk factor for late-onset AD, it would be interesting to perform a single-nucleus profiling analysis in APOE4 carriers to determine how this isoform impact the transcriptome of the various cell types, including microglia and astrocytes.

Single-cell profiling studies also suggest that the transcriptomic profile of astrocytes, like other cell types, might evolve with AD progression. Indeed, it was found that the high cell-type specificity for differentially expressed genes (DEGs) involved in apoptosis, autophagy, protein folding, and stress response in the early Braak stages lessens as the disease progresses (Mathys et al., 2019). Some gene expression variations observed in AD were also found to be common in multiple cell types. Glial cell types in general appear to decrease the activity of cell-death pathways especially at the beginning of the illness, and most of the cells that are still present in the late stage of AD had upregulated genes acting on misfolded proteins and cellular stress (Grubman et al., 2019). In the 5xFAD mouse model, it was also found that both disease-associated astrocytes (DAAs) and DAM shared a common transcriptional program involving multiple genes, including APOE and genes encoding Cathepsins B, D, and L involved in AD pathogenesis (Habib et al., 2020).

Many differences seem to exist between AD astrocytes in human and mouse models. For example, contrary to 5xFAD mice, astrocytes in AD human brain increased the expression of the complement factor 4 (C4B) and reduced the DAA marker SERPINA3 (Zhou et al., 2020). Moreover, the same study found that C4B and SERPINA3 were expressed in a different glial cell type (human astrocytes vs. mouse oligodendrocytes) (Zhou et al., 2020). In another report, the protein SERPINA3 and VIM, a marker of adult neurogenesis, have been shown to co-localize in hippocampal astrocytes near amyloid plaques in 5xFAD but not WT mice (Habib et al., 2020). Interestingly, they also found that DAAs start to appear before any memory impairment in 5xFAD mice, and that their abundance increases along disease progression as seen in AD patients as well as in normal aging of both WT mice and humans (Habib et al., 2020). These results are in line with another study showing that more astrocytes transition to a DAA state during aging in TauPS2APP mice, and that activated astrocytes are mostly found 10 to 20 microns away from amyloid plaques (Zeng et al., 2023). But as expressed above, while some changes are opposites or different between humans and other species, some changes are conserved. Further exploration of different *in vivo* and *in vitro* models will be needed to differentiate what causes these differences, how they interact with other factors of AD, and what their effects are on the progression of the disease.

## 4. Oligodendrocytes and OPCs

Oligodendrocytes are responsible for forming and repairing myelin, which enables rapid neuronal conduction in the central nervous system (CNS) (Kuhn et al., 2019). In humans, myelination is a highly regulated process that begins during gestation and continues through adulthood (Nave and Werner, 2014; Simons and Nave, 2015). Oligodendrocytes are generated from oligodendrocyte precursor cells (OPCs) from the germinal zones of the brain, including the subventricular zone and the subgranular zone (Maki et al., 2013). The OPCs proliferate in the germinal zones and then migrate into the cerebral cortex and pass through different stages of differentiation including the preoligodendrocytes until they become mature oligodendrocytes capable of forming the myelin sheath surrounding the axons (Maki et al., 2013; Marinelli et al., 2016). In addition to their role in myelination, oligodendrocytes participate in axonal survival through the production of lactate and growth factors including brain-derived neurotrophic factor (BDNF) and IGF-1 (Wilkins et al., 2001; Linker et al., 2010; Lee et al., 2012).

**FIGURE 3:**
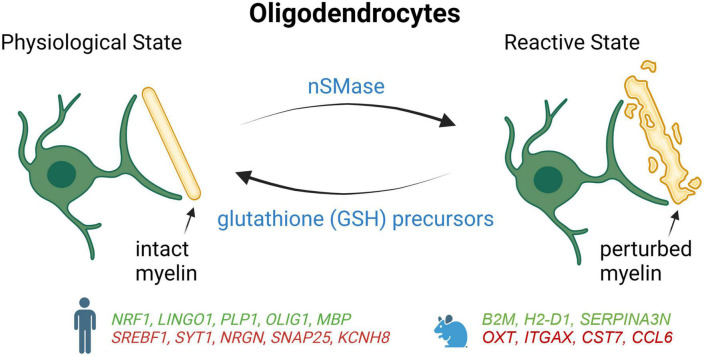
Schematic summary of oligodendrocyte states in AD patients and mouse models, which lead to degeneration of myelin. Genes upregulated (green) and downregulated (red) in snRNA-seq and snATAC-seq studies when comparing AD patients or 5xFAD mice with their respective controls. *NRF1*, Nuclear respiratory factor 1; *LINGO1*, leucine rich repeat and Ig domain-containing 1; *PLP1*, proteolipid protein 1; *OLIG1*, oligodendrocyte transcription factor 1; *MBP*, myelin basic protein; *SREBF1*, sterol regulatory element binding transcription factor 1; *SYT1*, synaptotagmin-1; *NRGN*, neurogranin; *SNAP25*, synaptosome associated protein 25; *KCNH8*, potassium voltage-gated channel subfamily H member 8; *B2M*, beta-2 microglobulin; *H2-D1*, histocompatibility 2, D region locus 1; *SERPINA3N*, Serine (or cysteine) peptidase inhibitor, clade A, member 3N; *OXTi*, oxytocin*i*; *ITGAX*, integrin alpha X; *CST7*, cystatin F; *CCL6*, chemokine (C-C motif) ligand 6.

As observed with microglia and astrocytes, a growing body of evidence suggests that oligodendrocytes also adopt a more reactive state during AD pathogenesis (Figure 3). Some studies have shown that toxic effects of Aβ in the AD brain can result in the loss of the OPCs and oligodendrocytes which may impair learning and memory (Xu et al., 2001; Desai et al., 2011). Single-nucleus profiling studies have shown that oligodendrocytes upregulated genes involved in myelination and differentiation in the entorhinal and prefrontal cortices of AD patients, such as LINGO1, ERBIN, BIN1, and CNTN2 (Grubman et al., 2019; Mathys et al., 2019). Conversely, another snRNA-seq study performed on dorsolateral prefrontal cortex tissue from AD brain reported a downregulation of genes responsible for myelination, differentiation, and axon guidance, while genes involved in lipid accumulation and oxidative stress were found to be turned up (Zhou et al., 2020). Thus, even if it is not clear at the moment why opposing changes occur, these results suggest that impaired axonal myelination and metabolic dysfunctions in AD oligodendrocytes could be an adaptive response to progressive neuronal losses observed over the course of the disease.

In a study combining snRNA-seq with snATAC-seq, it was found that the gene encoding the nuclear respiratory factor (NRF1), which regulates mitochondrial function, was upregulated in some oligodendrocyte subgroups of the prefrontal cortex in late-stage AD patients (Morabito et al., 2021). Thus, impaired mitochondrial function mediated by overexpression of NRF1 could lead to myelination disturbance and contribute to neuronal dysfunction in late-stage AD. Alterations in the *cis*-regulatory elements that control the expression of APOE and CLU were also detected in AD oligodendrocytes (Morabito et al., 2021). Moreover, the same study reported that the transcription factor SREBF1 had fewer accessible binding sites and a reduction of its expression, which correlated with lower levels of its target genes involved in the regulation of lipid metabolism and cholesterol (Morabito et al., 2021). Since Aβ has been shown previously to inhibit the activation of SREBF1 (Mohamed et al., 2018), amyloid accumulation could also contribute to the dysfunction of this transcription factor in AD.

As with AD astrocytes and microglia, differences have also been reported for oligodendrocytes between AD mouse model and human. In 5xFAD mice, mature oligodendrocytes adopt a reactive signature including Serpina3n and C4B near Aβ plaques that is not observed in human AD samples (Zhou et al., 2020). Since Serpina3n + oligodendrocytes are found to be partially dependent on TREM2, these results suggest that microglia activation is necessary to trigger reactive oligodendrocytes. Although oligodendrocytes also appear to be reactive in AD patients, these cells seem to upregulate a set of genes that is distinct from mice, including genes regulating oxidative, osmotic, and lipid metabolic pathways (Zhou).

Using a TauPS2APP mouse model, a disease-associated subtype of oligodendrocytes was found to be enriched around excitatory neurons containing high levels of hyperphosphorylated tau in the CA1 region of the hippocampus, especially at later stages when comparing 13–8 month-old mice (Zeng et al., 2023). The same study also reported that mature oligodendrocytes tended to concentrate in the 20–40 micron annulus of amyloid plaques, whereas OPCs mostly abound at 10–20 microns from plaques (Zeng et al., 2023). Altogether, these data suggest that DAM, which appear near plaque in the early stages of AD, participate in the activation of oligodendrocytes, OPCs, and DAAs that develop slightly farther from plaques and at later stages of the disease.

Another study discovered the association of Mbp + Cd74 + oligodendrocytes with AD’s progression in an APP knock-in AD mouse model using the droplet-based single-cell RNA sequencing at different time points over the course of AD (Park et al., 2023). The results also showed that the inhibition of the MAPK/ERK signaling can restore the impairment in myelination caused by the dysregulation of oligodendrocytes. Thus, the dysregulation patterns observed in the transcriptomic profiling of oligodendrocytes have shed light not only on the impairment of myelination due to the dysregulation of oligodendrocytes but also on how the imbalance in myelination can be restored by regulating signaling pathways such as the ERK signaling cascade with the potential to slow down the progression of the disease (Park et al., 2023).

## 5. Vascular cells and the blood-brain barrier

**FIGURE 4:**
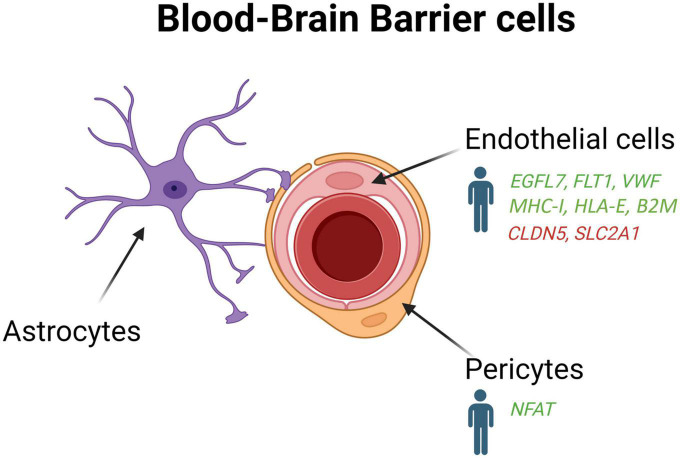
Schematic overview of the blood-brain-barrier cells (endothelial cells, pericytes and astrocytes) in AD patients and mouse models. Genes upregulated (green) and downregulated (red) in snRNA-seq and snATAC-seq studies when comparing AD patients or 5xFAD mice with their respective controls. *EGFL7*, EGF like domain multiple 7; *FLT1*, Fms related receptor tyrosine kinase 1; *VWF*, von willebrand factor; *MHC-I*, major histocompatibility complex I; *HLA-E*, major histocompatibility complex, class I, E; *B2M*, beta-2-microglobulin; *CLDN5*, claudin 5; *SLC2A1*, solute carrier family 2 member 1; *NFAT*, nuclear factor of activated T-cells.

The BBB plays an important role in protecting the brain from pathogens and toxins circulating in the blood and maintains homeostasis in the CNS (Daneman and Prat, 2015). It is a selective barrier which filters components such as oxygen, water, and nutrients necessary for physiological regulation of the brain while eliminating metabolic products generated by the parenchyma (Daneman and Prat, 2015). The BBB is located between the cerebral capillary blood and the interstitial fluid of the brain, and is made up of capillary endothelial cells, pericytes, and astrocytes (Figure 4; Engelhardt, 2003). Endothelial cells form tight junctions which limit the diffusion of bacteria, viruses, and large or hydrophilic molecules into the cerebrospinal fluid (CSF), and only allow select molecules to passively diffuse such as O_2_, CO_2_, and hormones. Endothelial cells can also actively transport substances such as glucose across the barrier using selective transporters.

Pericytes and astrocytes are also of great importance for the function and maintenance of the BBB (Daneman and Prat, 2015). Pericytes, contractile cells embedded into the basement membrane of small blood vessels, communicate with endothelial cells through direct physical contact and paracrine signaling to stabilize and control their maturation. Pericytes are integral for the neurovascular unit since they regulate capillary blood flow, facilitate the clearance and phagocytosis of cellular debris, and provide permeability in the BBB (Kadry et al., 2020). Although it is well established that BBB leakage, amyloid deposition in blood vessels, and cerebral amyloid angiopathy are major contributor of AD pathogenesis (Ellis et al., 1996; Johnson et al., 2007; Kisler et al., 2017), the molecular pathways underpinning these alterations still needs to be elucidated.

Among the non-neuron cellular populations in the brain, the role of vascular disturbances mediated by endothelial cells and the pericytes in the BBB is often overlooked in the context of AD. For a better understanding of the vascular properties of the brain, the profiling of the endothelial cells and the pericytes is an essential step to determine their interactions in the various states of physiology and pathology. Although vascular cell density (Niedowicz et al., 2014; Keller et al., 2018) approaches total glia density (Keller et al., 2018), many datasets have failed so far to identify transcriptome clusters associated specifically with pericytes (Grubman et al., 2019; Mathys et al., 2019; Lau et al., 2020; Zhou et al., 2020; Leng et al., 2021; Zeng et al., 2023), or have only captured low yields of endothelial cells (Grubman et al., 2019; Mathys et al., 2019; Habib et al., 2020; Zhou et al., 2020; Leng et al., 2021; Morabito et al., 2021; Zeng et al., 2023).

To enrich vascular and perivascular cell types for single-cell analysis, Yang et al. (2022) developed a new method to extract nuclei from microvessels from the hippocampus and cortex of AD tissue samples. They found that 30 of the 45 genes most tightly associated to AD risk by GWASs are expressed in the human brain vasculature. An important loss of brain nuclei across many vascular and perivascular cell types, including endothelial cells and pericytes, was also found in AD (Yang et al., 2022). They identified two subtypes of human pericytes, among which M-pericytes involved in extracellular matrix organization exhibited selective vulnerability in AD (Yang et al., 2022). Moreover, gene expression alterations in fibroblasts and mural cells also implicated dysregulated blood flow, which could account for the cerebral hypoperfusion observed in AD patients (Roher et al., 2012; Nortley et al., 2019). Interestingly, a pronounce interferon inflammation was found in the endothelial cells of APOE4 carriers, which display enhanced breakdown of the BBB before cognitive decline (Montagne et al., 2020). Human hippocampal pericytes from APOE4 carriers were also reported to exhibit an upregulation of the gene encoding nuclear factor of activated T cells (NFAT), which was found previously to be dysregulated in AD (Reese and Taglialatela, 2011).

In contrast to Yang et al. (2022) that did not identify novel vascular cell subclusters in the hippocampus and cortex of AD patients, Lau et al. (2020) observed the formation of a subpopulation of angiogenic endothelial cells in the prefrontal cortex of individual with AD. These angiogenic endothelial cells were characterized by an upregulation of genes involved in angiogenesis, including EGFL7, FLT1, and VWF, and antigen presentation such as MHC-I, HLA-E, and B2M. In the APP/PS1 mouse model, a regulator of insulin sensitivity (mNat1) was also shown to regulate endothelial cell necroptosis, Aβ deposition, and cognitive function (Zou et al., 2020). These data indicate that endothelial cells could contribute to angiogenesis abnormalities, decreased brain perfusion, and dysregulation of immune response in AD pathogenesis (Zlokovic, 2011; Bennett et al., 2018; Sweeney et al., 2018).

## 6. Perspective

Although the role of neuronal cells in AD pathogenesis is undeniable, the emergence of single-nucleus profiling over the last few years has also highlighted the critical impact of non-neuronal cells at all stages of the illness. These studies represent an incredibly rich dataset that can be mined to investigate novel hypotheses and characterize in a much broader manner the disease by integrating the influence of each cell type on AD pathology. Since major effort has been made to give open access to these large datasets, direct comparisons between different single-nucleus studies were conducted to give a more comprehensive view of the similarities and discrepancies that might exist between these data banks. This will help to better identify the most promising molecules that could have therapeutic benefits to prevent or halt the disease. It will also be important to pool these datasets to obtain enough cells or nuclei to achieve more consistent and meaningful results, especially for cell types that are less abundant such as microglia, or more difficult to isolate such as endothelial cells and pericytes. Combining datasets will also be valuable to better characterize sex and gender differences in AD by acquiring enough cells or nuclei from each group to perform reliable stratification analysis.

Since single-cell analysis is a relatively new field of investigation, the genetic signature of each cell type in the various brain regions during disease progression is still scattered. Although some studies have included a single-nucleus transcriptomic analysis at varying degrees of AD pathology in human and mouse model (Keren-Shaul et al., 2017; Mathys et al., 2019; Habib et al., 2020; Zeng et al., 2023), other studies have focused only on genes alterations of the different cell types in the late stages of the disease (Grubman et al., 2019; Lau et al., 2020; Zhou et al., 2020; Morabito et al., 2021; Anderson et al., 2023). Since the transcriptomic profile of each cell type evolves as the illness progress, determining which genes are differentially expressed in the preclinical or prodromal stages of AD in all cell types will be crucial to find novel therapeutic molecular targets effective before neurodegeneration causes irreversible brain damage and severe memory losses. Better understanding these changes will also be important for predicting potential side effects of AD treatments, as modeling how non-neuronal cells respond to changes in their environment can help us better evaluate how they may react to changes in their surroundings caused by pharmacological treatments.

Since the animal models available at the moment have failed to fully capture the broad spectrum of pathological events seen in AD patients (Ashe and Zahs, 2010; Brouillette, 2014), it is not surprising that many differences were observed between single-nucleus studies performed in mouse models and AD patients. The transcriptomic changes observed in human AD brain samples are the results of all the phenomena such as Aβ and tau pathologies, neurodegeneration and neuroinflammation that arise in a specific spatio-temporal manner. In this context, it is difficult to decipher which transcriptomic alterations are related to which neuropathological events in these human tissues. Thus, animal models developing solely Aβ pathology, for example after chronic injections in the hippocampus (Brouillette et al., 2012), could help untangling the molecular pathways affected exclusively by amyloid deposition in the brain. Since Aβ oligomers start to accumulate before the appearance of other pathological hallmarks in AD (Sperling et al., 2011; Brouillette, 2014), it would be very interesting to determine the specific impact of these oligomers at the transcriptomic and epigenetic levels to potentially find novel therapies that would prevent synapse and cell death in the early stages of AD. These experiments could also be useful to uncover new early biomarkers of the disease.

Since dissociation of intact cells is difficult to accomplish from archived, frozen post-mortem brain tissue, all human studies performed so far profile single nuclei instead of single cells. Thus, one non-negligible limitation of using a single nucleus profiling analysis is the loss of information regarding mRNA transcripts that translocated into the cytoplasm of the cell and that are not present anymore in the nuclei. Thus, this technical constraint could also induce discrepancies between transcriptome and proteome networks if both analyses cannot be performed in the whole cell.

The use of multi-omic approaches will also be needed to have a better view of the different facets of AD. With the occurrence of new technologies that allow to perform a transcriptomic and epigenetic analysis simultaneously within the same nuclei of each cell type as performed by Anderson et al. (2023), it is now possible to investigate changes in transcription factors that act on open chromatin nearby genes whose expression is altered in the same nucleus. Since the biological effectors of genetic risk factors in AD are mostly proteins and that not all mRNA transcripts correlate well with protein levels (Zhang et al., 2014; Mertins et al., 2016; Johnson et al., 2022), the next great challenge will be to determine if changes in gene expression reflect on the level of proteins at the single-cell resolution.

## 7. Conclusion

The recent development of single cell approaches has allowed us to enter a new era of knowledge that will bring unprecedented information on the fate of neuronal and non-neuronal cells in AD. If non-neuronal cells have been overlooked over the years in the field of AD, the advent of these unbiased methods has highlighted their critical contribution on the progression of the disease. Since all cell types are interconnected, looking at them as a whole is essential to better understand the impact of each neuropathological event that takes place in the brain of AD patients. Determining the transcriptomic profile of each cell type is an important step which will help to uncover cellular and molecular pathways underlying neurodegeneration and cognitive decline over the course of the disease. Combining this approach with epigenomic and proteomic analyses at the single cell level in various stages of the disease and brain regions will give a clearer picture of the different aspects of AD. Hopefully, this will pave the way in finding new therapeutic targets to prevent the onset of this neurodegenerative disease.

## Author contributions

T-MV, VH, AKU, DL-K, and JB wrote, revised, and edited the manuscript. All authors approved the final version of the manuscript.
